# Biomaterials in organoid research: current state and future directions

**DOI:** 10.3389/fbioe.2026.1895558

**Published:** 2026-08-21

**Authors:** Laura Klasen, Ramin Nasehi, Lennart Selzener, Laura De Laporte

**Affiliations:** 1 DWI—Leibniz Institute for Interactive Materials, Aachen, Germany; 2 Institute for Technical and Macromolecular Chemistry, RWTH Aachen University, Aachen, Germany; 3 Department of Advanced Materials for Biomedicine (AMB), CBMS—Center for Biohybrid Medical Systems, AME—Institute of Applied Medical Engineering, RWTH Aachen University, Aachen, Germany

**Keywords:** biochemical cues, biomaterials, biophysical cues, natural matrices, organoids, synthetic matrices, technological advances

## Abstract

Organoid research has fundamentally reshaped *in vitro* approaches to modeling disease, drug response, and developmental processes. While the potential is great, the technology is limited by reproducibility and physiological accuracy challenges that arise partly from the shortcomings in extracellular matrix mimicking biomaterials that influence morphogenesis, differentiation, and functionality. In recent years, biomaterials for organoid systems have developed from biologically derived but poorly defined matrices toward tunable, dynamic, and modular systems that allow for precise control and better reproducibility of the microenvironment. This Mini-Review summarizes recent advances, with a focus on the last 3 years, in natural, synthetic, and hybrid biomaterials, highlighting engineered ECM–derived hydrogels, modified natural polymers, and synthetic systems with tunable viscoelasticity, degradability, and bioactive components. Furthermore, emerging trends and technological integrations, comprised of 3D and 4D bioprinting, granular hydrogels, organ-on-a-chip platforms, and AI-driven methods, will be discussed, which together support scalable and data-driven optimizations in organoid research. Summarized, these developments demonstrate the transition from a generic matrix-based culture toward engineered, tunable, and dynamic microenvironments, demonstrating biomaterial design as a fundamental element for next-generation organoid systems.

## 1 Introduction

Organoids have emerged as a groundbreaking tool for biomedical research. They are stem cell-derived three-dimensional structures that self-organize into tissues, recapitulating the architecture and functions of target organs. Thereby, they provide a powerful tool for developmental biology and organogenesis, disease modeling, drug discovery, screening and toxicology, and regenerative medicine and personalized therapy. Organoids differ from 2D and simple 3D culture models in structure, complexity, cellular architecture and function, reproducibility, and scalability. 2D cultures often lack complexity and spatial interactions, while simple 3D cultures lack relevant spatial organization due to their limited number of cell types and restricted structural organization of the cells. Organoids offer a high biological complexity by self-organization into functional tissue architectures, including specialized cell types, tissue-layering, and lumen formation (Sato et al., 2009; Lancaster et al., 2013). Even though organoids are among the most physiologically relevant *in vitro* systems, their widespread use for high-throughput applications like drug screening is still limited, due to challenges in standardization and scalability (At et al., 2025; Wang et al., 2025). These limitations arise due to the high sensitivity regarding growth conditions, which require precise control over culture conditions and the organoid’s microenvironment (Zhao et al., 2022). Organoid formation and differentiation are highly dependent on the surrounding matrix, which ideally should mimic the tissue-specific ECM as closely as possible. The key ECM parameters influencing tissue formation can be divided into two groups: biochemical cues, like integrin-binding motifs or growth factor release, and biophysical cues, like matrix stiffness, viscoelasticity, degradability, and porosity. Due to the central role of the ECM for tissue and thereby organoid formation, increasing attention has turned to biomaterial design as a strategy to improve organoids in terms of maturation, functionality, and reproducibility. The currently most abundant matrix used for organoid cultivation is Matrigel. Due to its natural origin, it is rich in biological cues, which enable great guidance and cell interaction important for organoid formation (Hughes et al., 2010). Despite their advantages, the use of Matrigel or other naturally-derived matrices like collagen or decellularized ECM is associated with several trade-offs. High batch-to-batch variabilities, undefined and complex composition, limited tunability, and tumor-derived origin result in poor reproducibility and suitability for quantitative studies, high-throughput screening, and good manufacturing practice (GMP) compatibility (Jeon et al., 2022). This creates a demand for alternative biomaterials capable of recapitulating the diversity of the tissue-specific ECM, while allowing for mechanistic insights and translational relevance (Gjor et al., 2016; Singh, 2017; Gjorevski et al., 2022). Engineered and synthetic matrices respond to these demands by providing a defined and controllable microenvironment that enhances reproducibility in organoid culture, thereby reducing batch-to-batch variability with a consistent chemical composition (Mulero-Russe and García, 2024; Kratochvil et al., 2019; Aisenbrey and Murphy, 2020). Their modular design enables tunability of mechanical and biophysical cues like stiffness, degradability, and ligand density, allowing for systematic investigation of specific parameters and their influence on organoid development and function (Hunt et al., 2021). Synthetic biomaterial platforms, e.g., polyethylene glycol-based (PEG) hydrogels, are scalable, which facilitates standardization and high-throughput production, supporting large-scale studies. A wide variety of engineered biomaterials has been developed, including dynamic and responsive matrices, disease-specific matrices, and integration with bioengineering technologies like microfluidics and bioprinting (Cruz-Acuña et al., 2017; Chaudhuri et al., 2016; Kozlowski et al., 2021). While these biomaterials pose great potential for a translational future of organoids, there are still challenges that need to be addressed (Kratochvil et al., 2019; Shao et al., 2025). Synthetic matrices often lack tissue-specificity due to their lower biological activity compared to natural matrices. To tackle these limitations, many new trends are emerging in the field of engineered biomaterials for organoid technologies.

This mini-review will provide an overview of the role of biomaterials as a microenvironment for organoid culture, with a focus on the most recent advances in biomaterial-based organoid platforms. It will highlight the trend towards specific engineered organoid niches, dynamically changing matrices, and high-throughput requirements.

## 2 Natural versus synthetic biomaterials for organoid culture

The recent developments in biomaterials for organoid culture demonstrate the increasing focus on defined and tunable matrices to enable mechanistic and translationally relevant model systems.

### 2.1 Natural biomaterials–engineered ECM complexity

Biologically-derived matrices offer a high intrinsic bioactivity, due to their adhesive ligands, growth-factor binding sites, and protease-sensitive domains. Materials like Matrigel, a tumor-derived matrix possesses mechanistic advantages providing basement-membrane components, such as laminin and collagen IV, as well as supplying a growth factor reservoir and a cell-remodellable network (Gjor et al., 2016). However, even though Matrigel and other natural polymers remain widely used, they also have their limitations, e.g., batch variability and limited chemical definition and tunability. Therefore, generic universal matrix platforms, such as Matrigel, are sought to be replaced with tissue-specific and tunable systems that recapitulate the bioactivity characteristics of the natural matrices. Current approaches increasingly focus on engineered natural biomaterials like decellularized extracellular matrices (dECM) (Li C. et al., 2024; Kim J. W. et al., 2022; Kim S. et al., 2022; Giobbe et al., 2019) and hybrid natural–synthetic matrices (Cho et al., 2025). A fully patient-derived model of ovarian cancer was established by combining patient-derived organoids together with patient-derived dECM, achieving a chemotherapeutic response similar to *in vivo* (Sensi et al., 2025). In another study, region-specific brain dECM is produced, promoting neural maturation in *in vitro* stroke models (Reginensi et al., 2025). These new approaches focus on harnessing the tissue-specific potential of natural matrices while minimizing allogeneic effects and artificial cell–matrix cross-talk, inherent to generic natural matrices. This reflects the current trend of leveraging the bioactivity of natural matrices while employing controlled mechanics to improve their use for developmental and disease modeling applications.

### 2.2 Synthetic biomaterials–defined, dynamic, and modular platforms

Synthetic biomaterials can be made of polymers (e.g., PEG, polyacrylamide) or natural macromolecules that are synthesized or produced in the lab (e.g., DNA, peptides, recombinant proteins). These molecules are chemically defined, allowing them to provide precise control over material properties. Therefore, they enable the mechanistic study of cell-matrix interactions and the influence of specific microenvironmental parameters on organoid development and functionality. The recent trend demonstrated a shift from static scaffolds to more advanced dynamic materials (Chrisnandy et al., 2022). Synthetic hydrogels with dynamic mechanical properties can tailor the viscoelasticity, stress-relaxation, and dynamic matrix stiffening/softening. Viscoelasticity describes the time-dependent mechanical characteristics of a matrix. Stress relaxation refers to the loss of stress under a constant deformation with relaxation times ranging from seconds to several hours (Lai et al., 2025; Ruiter et al., 2022), and matrix stiffnesses ranging from hundreds of pascals to several kilopascals. These characteristics are advantageous in guiding organoid morphology, lineage specification, and cell polarization (Xie et al., 2025; Soliman et al., 2024), by influencing cell-mediated matrix remodeling and force dissipation behavior. One example of dynamic synthetic hydrogels is DNA-encoded hydrogels with tunable viscoelasticity and stimuli-responsiveness (Wu et al., 2025). Dynamically crosslinked DNA matrices enabled variable stress-relaxation times, self-healing, and controllable degradation, allowing enhanced growth and morphogenesis of multiple organoid systems (Peng et al., 2023). In a similar approach, a DNA-encoded bioink enabled matrix remodeling, resulting in enhanced chondrocyte differentiation and zonal organization in cartilage organoids (Chen et al., 2025). While DNA-based matrices are widely researched, there is also a multitude of alternative supramolecular hydrogels, such as the UPy-IKVAV hydrogels that are based upon a ureido-pyrimidone moiety with adjustable stiffness, ligand type, and concentration (Rijns et al., 2026). Using these hydrogels, a mechanistic study of guided intestinal organoid polarity was performed, revealing that the laminin-mimicking IKVAV sequence is essential for guiding correct basal-out organoid polarity in fully synthetic hydrogels. Furthermore, synthetic methacrylated hyaluronic acid hydrogels demonstrated the positive impact of viscoelasticity on spinal cord organoid patterning and vascularization through co-culture with blood-vessel organoids, revealing that stress-relaxing environments promote regional patterning and nuclear Yes-associated protein (YAP) translocation (Chen et al., 2024). Most of the above-described hydrogels express stimuli-responsiveness to biochemical and biophysical stimuli, in addition to their dynamic nature, creating an adaptive environment that is able to be shaped during organoid growth (Neumann et al., 2023; Acharya et al., 2025). Besides growing organoids in bulk hydrogels, a recent trend shifted towards hybrid or modular systems that apply microgels or hydrogel units to generate spatial heterogeneity, e.g., in stiffness or biochemical cues, or to support patterned differentiation and organoid assembly (Blatchley and Anseth, 2023; Vaziri et al., 2026; Klasen et al., 2025; Eiken et al., 2025), which will be elaborated on in Chapter 4.3.

## 3 Biophysical and biochemical cues in organoid development

**FIGURE 1:**
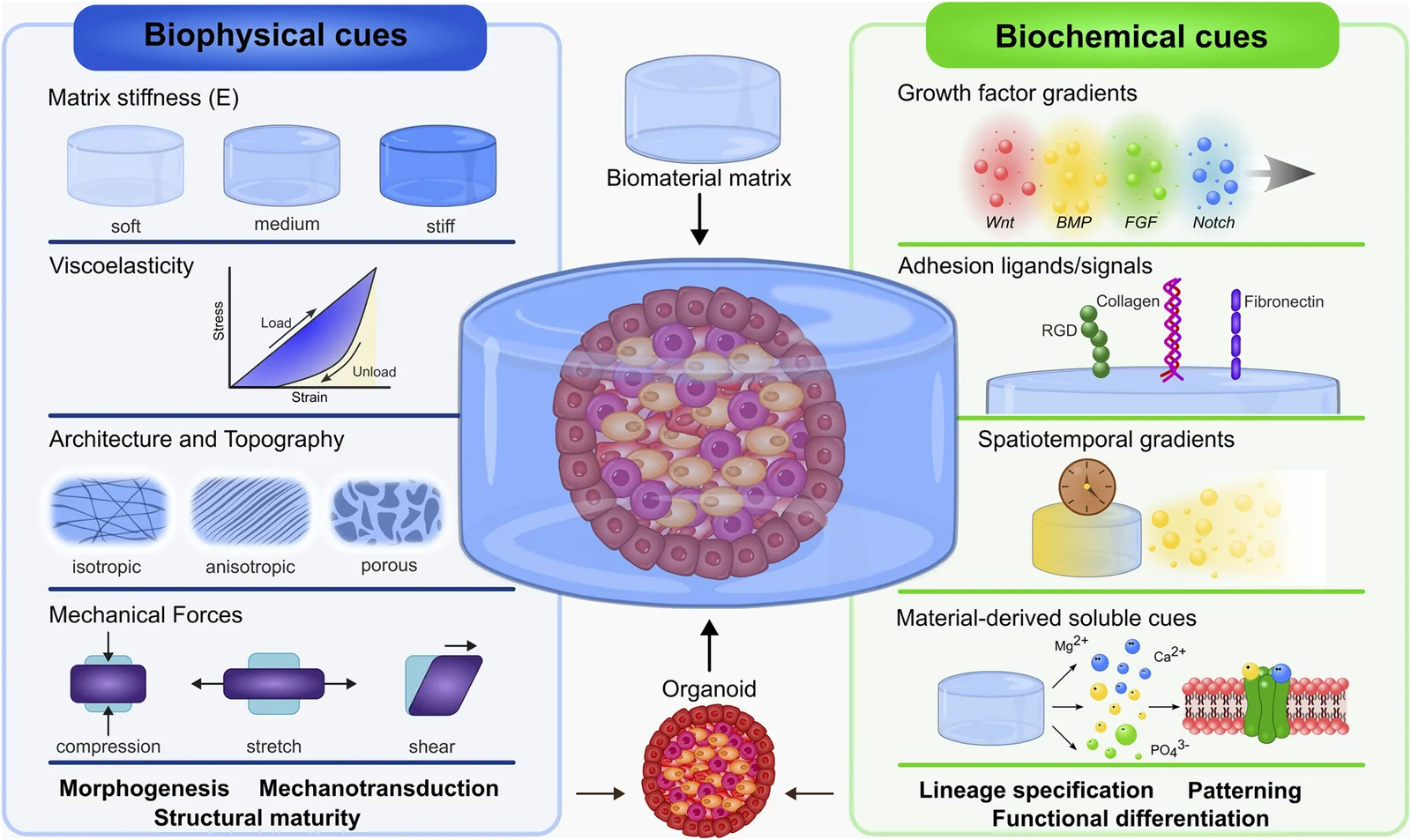
Schematic overview of biophysical and biochemical cues of biomaterials. The schematic was prepared with Inkscape.

A remaining challenge in organoid development remains the generation of functional organoids that correctly mimic the structure and function of their respective tissues *in vivo*. To improve this process, the understanding of the importance of biophysical and biochemical cues for organoid differentiation and maturation is crucial (Figure 1).

### 3.1 Biophysical cues

The biophysical cues of the microenvironment can be divided into mechanical and architectural/topographical cues; however, these factors usually occur in parallel and show symbiotic effects. In *in vivo* cell niches, ECM is continuously deposited, degraded, and reshaped, changing the mechanical properties of the matrix during tissue development. Therefore, mechanically dynamic matrices are vital for organoid fate decisions by mimicking the evolving niches through development, allowing for stage-specific morphogenesis and maturation. Through the promotion of physiologically relevant differentiation pathways and functional phenotypes, these materials better enable mechanistic studies and translatability (Blache et al., 2022). Parallel to the niche-specific dynamics, the mechano-transduction via YAP/Transcriptional coactivator with PDZ-binding motif (TAZ) pathways and integrin signaling are fundamental and can be positively influenced through viscoelastic and stress-relaxing materials (Huang et al., 2025; Dupont et al., 2011). These matrices have promoted lumen formation, branching, and lineage commitment in different organoids (Atcha et al., 2023).

The matrix microarchitecture and topography have been proven to beneficially impact organoid culture through the regulation of organoid shape, nutrient diffusion, and spatial organization guidance (Qazi et al., 2022). One of the milestones in this field was the report that spatial confinement within a hybrid collagen-I/Matrigel hydrogel scaffold with laser-ablated microcavities guides the self-organization of intestinal stem cells (ISCs) into a functional, tube-shaped intestinal epithelium (Nikolaev et al., 2020). The confinement promoted the *in vivo*-like spatial arrangement of crypt- and villi-like structures. Recent research explored these topographical cues further; applying micropatterned geometries made it possible to predict the place and time where specific tissue patterns of intestinal stem cells form by manipulation of the physical environment and geometry alone (Gjorevski et al., 2022). To achieve this, photosensitive PEG-based hydrogels were locally softened when exposed to light to generate controlled crypt-like budding at these softened areas, which led to the formation of functional crypts.

### 3.2 Biochemical cues

Engineered biomaterials can provide biochemical cues that guide organoid development, often acting in parallel with the biophysical cues. The most abundant method to incorporate these cues into the matrix is tethering of active ligands, growth factors, or the use of active material building blocks. Recent work described the use of the integrin-activating protein invasin as a defined and animal-free matrix alternative to Matrigel (Wijnakker et al., 2025). The authors state that the intracellular signals generated by the interaction of invasin with multiple heterodimeric integrin receptors favor organoid development comparable to that in complex biological matrices, demonstrating the importance of investigating the effects of specific biochemical cues. These cues can also be applied to generate defined complex tissue niches by engineering synthetic biomaterials with tissue-specific bioactive compounds. As an example, epithelial-secreted ECM was incorporated into synthetic PEG hydrogels, promoting organoid survival, single-cell survival of intestinal stem cells without exogenous Wnt antagonists, and multicellular patterning (Chrisnandy and Lutolf, 2025). Apart from these methods, it is also possible to harness the bioactivity of purely synthetic materials to improve the development of certain organoids. A chemically defined matrix with incorporated calcium silicate nanowires and magnesium silicate nanospheres, demonstrating the potential of silicate biomaterials to promote functional maturation of bone marrow organoids (Ma et al., 2025). Through the release of bioactive ions (Ca^2+^, Mg^2+^, SiO_4_^4-^), enhanced endothelial network formation, mesenchymal stem cell renewal, and the upregulation of osteogenic and angiogenic genes were observed. Recent developments also apply spatiotemporally engineered release of biochemical cues (Afting et al., 2024), which often comes with dynamic biophysical changes through degradable crosslinks (Soliman et al., 2024; Kharkar et al., 2013) or biochemical gradient formation (Zhang et al., 2022). These advances can be particularly important in the context of vascularization, in which matrix stiffness, degradability, adhesive ligands, and growth-factor availability together regulate microvascular network formation (Brown et al., 2020). An example describes PEG/gycosaminoglycan (GAG) matrices where the GAG concentration and the sulfation pattern can tune pro-angiogenic signaling, through the availability and activity changes in GAG-binding growth factors, thereby underlining the ability of synthetic biomaterials to guide vascularization (Limasale et al., 2024). On the other hand, enzymatically degradable crosslinks enable cell-mediated remodeling and dynamic exposure to embedded cues, while biochemical gradient formation within the matrix allows for regional specificity in organoids, demonstrating the need for an interplay of biophysical and biochemical cues to create *in vivo*-mimicking tissue niches, while supporting vascular integration and maturation in these tissues (Cao et al., 2021; Broguiere et al., 2018).

## 4 Technological integration

**FIGURE 2:**
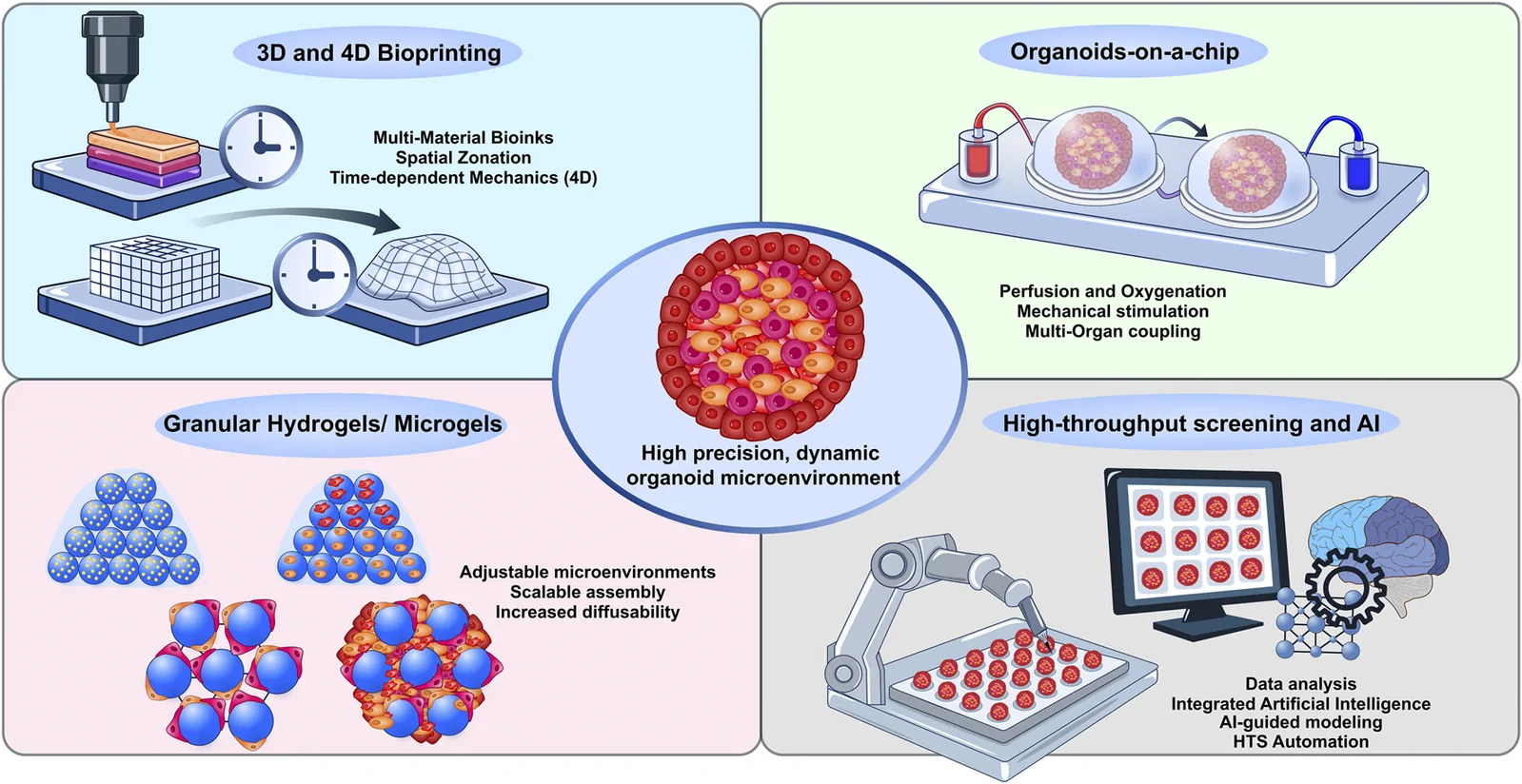
Schematic overview of emerging trends and future perspectives. The schematic was prepared with Inkscape.

To enable the production of high-precision, high-throughput, and adaptive organoid platforms, close integration of biomedical research with engineering, chemical, computational, and physical sciences is required. Amid these technological advances, material choice and design remain critical parameters to be assessed. By leveraging the advantages of advancements in biomaterials, fabrication techniques, computational tools, and robotic technologies, emerging organoid production and processing technologies have appeared (Yi et al., 2021) (Figure 2).

### 4.1 3D and 4D bioprinting

Recent developments in organoid bioprinting demonstrate the potential of combining biomaterial research and bio-manufacturing as evidenced by the fabrication of architecturally engineered model systems that exhibit structural and spatial precision (Li et al., 2025; Bittner et al., 2018; Homan et al., 2016). Bioprinting approaches allow for multi-material use, high-resolution patterning, and spatially precise arrangements of materials and cells, thereby creating *in vivo*-like tissue morphologies, which result in improved functionality and cellular composition in organoids (Khanna et al., 2022; Layrolle et al., 2022; Huan et al., 2025; Zhang et al., 2024; Lawlor et al., 2021). A recently developed embedded bioprinting approach demonstrated that induced pluripotent stem cell (iPSC)-laden bioinks printed into granular support hydrogels can undergo viscoelasticity-dependent shape morphogenesis and timed WNT-driven cardiac differentiation (Pramanick et al., 2026). Furthermore, pancreatic progenitor cells, mesenchymal stem cells (MSCs), and endothelial cells were printed by extrusion in a fibrin bioink, supplemented with gelatin and HA, demonstrating mechanical and spatial regulation of cell fate (Edri et al., 2024). They observed stiffness-dependent differentiation into endocrine and exocrine cells, as well as self-organization of branched vascular networks. Another recent study elucidated the synergy between geometry and biochemical cues by using an in-bath bioprinting technique to print spatially patterned pancreatic islet peripheries, using a peri-islet niche-like bioink (PINE) (Kim et al., 2025). They applied the geometrical guidance of the bioprinting process together with patterning islet cells within a 50 µm distance from endothelial cells, thereby achieving increased cell interaction, vascularization, and maturation. Bioprinting, furthermore, facilitates the production of large-scale organoids (Brassard et al., 2021; Wang J. et al., 2024). Bone organoids of 5–10 mm in diameter were generated using a hydroxyapatite hybrid bioink with an intrinsic self-mineralizing quality, which made the long-term cultivation and progressive maturation of bone organoids possible. 4D bioprinting, which applies spatial-temporally active biomaterials as bioinks, adds a new dimension to conventional bioprinting (Mierke, 2024; Arias-Peregrino et al., 2025; Lai et al., 2024). This method harbors prospects like dynamic adaptation to cellular processes (Douillet et al., 2022), enhanced functionality through spatiotemporal drug or growth factor release, or improved surgical fitting. The bioprinting approaches currently still face many challenges, such as ensuring sufficient vascularization for large-scale constructs, bioink limitations for printability, scalability, and translational integration, which need to be addressed in the future (Li et al., 2025).

### 4.2 Organ-on-a-chip systems

The integration of organoid technology with organ-on-a-chip methods holds great potential to enhance the controlled perfusion, oxygenation, and mechanical stimulation of organoids, thereby facilitating vascularization and reducing necrotic core formation. Through this dynamic stimulation, organoid differentiation can be positively influenced (Byeon et al., 2024; Cho et al., 2021), while the setup also allows for multi-organ coupling (Novak et al., 2020; Maschmeyer et al., 2015), posing important systemic test platforms for drug testing and disease modeling (Z et al., 2024). Furthermore, the prospect of perfusable vascularized organoids would be beneficial for long-term cultivation, allowing for advanced functionality and maturity, as well as developmental studies (Wang et al., 2023; Zhang et al., 2021; Li Z. et al., 2024; De L et al., 2024; Quintard et al., 2024). The requirements for materials used in these setups are complex, as the materials need to be compatible with the technological setup, while also being biologically active, favoring organoid development. In addition to biocompatibility, biomimicry, and mechanobiological properties, these platforms also require specific technological and physical aspects. The material needs long-term mechanical stability, chemical resistance against absorption of small hydrophilic molecules, gas permeability to ensure oxygen diffusion, suitable methods for sterilization, and compatibility with microfabrication techniques like lithography. Common practice is a combination of mechanically stable scaffolding materials like polydimethylsiloxane (PDMS) or alginate, functionalized with a bioactive coating, with natural materials like Matrigel or collagen (Ramírez-González et al., 2025). In one example, an alginate-chitosan-dECM composite was established to grow chondrocytes and MSCs into an articular cartilage model (Upadhyay et al., 2024). The alginate was used to produce the structural scaffold, while chitosan provided cell-adhesion functionality, and the dECM created a niche-specific microenvironment. This matrix significantly enhanced the phenotypic and genotypic expression of cartilage-specific markers (such as collagen type II and aggrecan) in native and stem-cell-induced chondrocytes, thereby generating a functional hyaline-type cartilage niche. Other applications for microfluidic systems are microfluidic hydrogel-droplet approaches, extending the concept towards embedded organoid-specific workflows. This was recently demonstrated for the high-throughput generation of intestinal organoids in uniform hydrogel droplets, creating small material/organoid constructs, improving scalability, reproducibility and compatibility with downstream screening (Lavick et al., 2026).

### 4.3 Granular hydrogels and microgel systems

An emerging trend holding great potential for organoids is the use of granular hydrogels or microgel systems. The term granular hydrogels can describe a variety of systems, like microporous annealed particle scaffolds (MAPs) (Griffin et al., 2015), jammed granular hydrogels, or sacrificial granular hydrogels (Vaziri et al., 2026; Qazi and Burdick, 2021). These systems offer a variety of advantages for tissue engineering applications by enabling enhanced mass transport and diffusion, endogenous cell infiltration allowing for dense tissues, superior injectability, printability, and modularity that allows for heterogeneity (Segura, 2024; Joshi et al., 2025). These novel materials are compatible with other emerging technologies like 3D and 4D bioprinting (Nakamura et al., 2024). In a multi-material 4D printing technique, temperature-responsive granular hydrogel inks, composed of norbornene-modified hyaluronic acid (NorHA) microgels, crosslinked with dithiol-functionalized poly (N-isopropylacrylamide), were printed into a non-responsive granular hydrogel support bath. The resulting structure was further stabilized through visible-light-mediated post-crosslinking of the microgels. They furthermore incorporated microgels with different transition temperatures into one construct, allowing for spatial patterning and sequential multi-stage shape changes, like bending. The macroporosity of the granular system decreased the response time of temperature-induced volume phase transitions by 20-fold, compared to non-porous bulk hydrogels. So far, this system has not yet been reported with cells. While beneficial for many applications, the use of interlinked microgels as granular scaffolding materials has a limitation for tissues with complex structures or developmental processes like organoids. The chemical interlinking limits the cellular self-organizing potential, causing recent research to focus on cell-guided interlinking of microgels through self-assembly (Claxton et al., 2024). This technology was used to generate iPSC-based organoids that combine stem cell expansion and differentiation in one construct (Klasen et al., 2025). The injectability of the microgel/cell suspension allows for a unified workflow, which was demonstrated to be automation-compatible and scalable, while also posing the other advantages of granular biomaterials, such as enhanced diffusion kinetics through the microgels. Although many granular hydrogels currently focus on general tissue-engineering applications and concepts, the recently emerging organoid applications indicate the support of these materials regarding iPSC expansion, differentiation, and scalability of organoid workflows (Klasen et al., 2025). Granular hydrogels, thereby, hold a great potential for future developments in organoid research, when their porosity, modularity, and handling advantages are matched to the specific biological requirements, especially in the context of scalable, spatially patterned, and modular culture setups.

### 4.4 Artificial intelligence and high-throughput screening

The need for increased translatability and, thereby, large-scale production of organoid models is widely recognized. As the organoid model complexity and the throughput increase, massive volumes of imaging, omics, and biophysiological data are generated, which require extensive computational and data handling (Wang H. et al., 2024). The combination of high-throughput screening (HTS) technologies and artificial intelligence (AI) methods, like convolutional neural networks (CNNs), enables the automated segmentation, feature extraction, and temporal tracking of organoids, as well as the correlation of different data types, thereby allowing the conversion of complex biological phenotypes into readouts for biomaterial screening (Park et al., 2023; Qin et al., 2025). Beyond data analysis, AI-driven predictive modeling is used to anticipate organoid growth, morphogenesis, material-tissue interactions, and developmental changes by linking specific material variables to biological changes. This helps to prioritize the material conditions expected to support a specific organoid phenotype through *in silico* experimental design that causes a significant reduction in empirical screening (Bai et al., 2024; Maramraju et al., 2024). Furthermore, AI-based tools can enable automated quality control through the early identification of abnormal organoids, thereby enhancing reproducibility, minimizing experimental variability, and reducing overall costs. In addition, AI can integrate and correlate multimodal datasets, such as imaging, molecular, and functional readouts, to identify hidden patterns, uncover relationships between different biological parameters, and generate predictive models that improve our understanding of organoid development and function. To train and validate these models, scientists rely on large, well-curated datasets that capture the variability across culture conditions, organoid phenotypes, and material influences, highlighting the importance of standardized, high-quality data repositories (Schröter et al., 2024; Rozenblatt-Rosen et al., 2017; He et al., 2024). The integration of AI analytics and predictive modeling poses a more efficient discovery of biomaterials for specific organoid applications, accelerating the engineering of novel, improved material systems. To be able to use these methods, there is a need to generate large datasets of biomaterial-organoid interactions, requiring HTS-compatible, scalable, and cost-effective materials for the future to exploit the full potential of computational advances.

## 5 Conclusion

The recent trends in biomaterials for organoid culture demonstrate a shift from static, empirically designed matrices toward chemically defined, dynamic, and adaptive systems. Naturally-derived matrices still remain important due to their high bioactivity, while synthetic and hybrid systems are gaining increasing popularity due to their precision in controlling mechanical, biochemical, and architectural parameters. Combining the developments in both natural and synthetic materials, the generation of dynamic feedback-responsive microenvironments that closely mimic the ECM niche enables the more accurate recapitulation of organoid development and differentiation. The integration of these materials with emerging technologies such as 3D and 4D bioprinting, organ-on-a-chip platforms, and AI-driven design will be beneficial to further drive organoid research in the future, bringing research closer to translation in personalized medicine and drug discovery. While there exist a variety of challenges for realizing such applications, e.g., HTS-compatible biomaterial design, large data storage and 3D/4D analysis, or creation of physiologically relevant functional organoids, there is a huge potential for interdisciplinary work to accelerate organoid research and discover solutions. Future directions will likely incorporate closed-loop systems, combining material properties, cell-material interactions, and predictive computational/AI-driven models to unite tailored materials, spatio-temporal control, and data-driven optimization to push many organoid models into translatability.

## References

[B1] Acharya R. Dutta S. D. Mallik H. Patil T. V. Ganguly K. Randhawa A. (2025). Physical stimuli-responsive DNA hydrogels: design, fabrication strategies, and biomedical applications. J. Nanobiotechnol. 23 (1), 233. 10.1186/s12951-025-03237-w PMC11929200 40119420

[B2] Afting C. Walther T. Drozdowski O. M. Schlagheck C. Schwarz U. S. Wittbrodt J. (2024). DNA microbeads for spatio-temporally controlled morphogen release within organoids. Nat. Nanotechnol. 19 (12), 1849–1857. 10.1038/s41565-024-01779-y 39251862 PMC11638066

[B3] Aisenbrey E. A. Murphy W. L. (2020). Synthetic alternatives to matrigel. Nat. Rev. Mater 5 (7), 539–551. 10.1038/s41578-020-0199-8 32953138 PMC7500703

[B4] Arias-Peregrino V. M. Tenorio-Barajas A. Y. Mendoza-Barrera C. O. Román-Doval J. Lavariega-Sumano E. F. Torres-Arellanes S. P. (2025). 3D printing for tissue engineering: printing techniques, biomaterials, challenges, and the emerging role of 4D bioprinting. Bioeng. (Basel) 12 (9), 936. 10.3390/bioengineering12090936 PMC12467047 41007181

[B5] Atala A. Bischof J. C. Chen C. S. Fisher J. P. Hermanson D. L. Howe C. L. (2025). The need for an organoid manufacturing, preservation, and distribution center. Stem Cells Transl. Med. 14 (7), szaf031. 10.1093/stcltm/szaf031 40627335 PMC12236065

[B6] Atcha H. Choi Y. S. Chaudhuri O. Engler A. J. (2023). Getting physical: material mechanics is an intrinsic cell cue. Cell Stem Cell 30 (6), 750–765. 10.1016/j.stem.2023.05.003 37267912 PMC10247187

[B7] Bai L. Wu Y. Li G. Zhang W. Zhang H. Su J. (2024). AI-enabled organoids: construction, analysis, and application. Bioact. Mater. 31, 525–548. 10.1016/j.bioactmat.2023.09.005 37746662 PMC10511344

[B8] Bittner S. M. Guo J. L. Melchiorri A. Mikos A. G. (2018). Three-dimensional printing of multilayered tissue engineering scaffolds. Mater Today Kidlingt. 21 (8), 861–874. 10.1016/j.mattod.2018.02.006 30450010 PMC6233733

[B9] Blache U. Ford E. M. Ha B. Rijns L. Chaudhuri O. Dankers P. Y. W. (2022). Engineered hydrogels for mechanobiology. Nat. Rev. Methods Prim. 2 (1), 98. 10.1038/s43586-022-00179-7 PMC7614763 37461429

[B10] Blatchley M. R. Anseth K. S. (2023). Middle-out methods for spatiotemporal tissue engineering of organoids. Nat. Rev. Bioeng. 1 (5), 329–345. 10.1038/s44222-023-00039-3 37168734 PMC10010248

[B11] Brassard J. A. Nikolaev M. Hübscher T. Hofer M. Lutolf M. P. (2021). Recapitulating macro-scale tissue self-organization through organoid bioprinting. Nat. Mater 20 (1), 22–29. 10.1038/s41563-020-00803-5 32958879

[B12] Broguiere N. Isenmann L. Hirt C. Ringel T. Placzek S. Cavalli E. (2018). Growth of epithelial organoids in a defined hydrogel. Adv. Mater 30 (43), e1801621. 10.1002/adma.201801621 30203567

[B13] Brown A. He H. Trumper E. Valdez J. Hammond P. Griffith L. G. (2020). Engineering PEG-based hydrogels to foster efficient endothelial network formation in free-swelling and confined microenvironments. Biomaterials 243, 119921. 10.1016/j.biomaterials.2020.119921 32172030 PMC7203641

[B14] Byeon J. H. Jung D. J. Han H.-J. Son W.-C. Jeong G. S. (2024). Fast formation and maturation enhancement of human liver organoids using a liver-organoid-on-a-chip. Front. Cell Dev. Biol. 12. 10.3389/fcell.2024.1452485 PMC11349704 39206088

[B15] Cao H. Duan L. Zhang Y. Cao J. Zhang K. (2021). Current hydrogel advances in physicochemical and biological response-driven biomedical application diversity. Signal Transduct. Target. Ther. 6 (1), 426. 10.1038/s41392-021-00830-x 34916490 PMC8674418

[B16] Chaudhuri O. Gu L. Klumpers D. Darnell M. Bencherif S. A. Weaver J. C. (2016). Hydrogels with tunable stress relaxation regulate stem cell fate and activity. Nat. Mater 15 (3), 326–334. 10.1038/nmat4489 26618884 PMC4767627

[B17] Chen X. Liu C. McDaniel G. Zeng O. Ali J. Zhou Y. (2024). Viscoelasticity of hyaluronic acid hydrogels regulates human pluripotent stem cell-derived spinal cord organoid patterning and vascularization. Adv. Healthc. Mater 13 (32), e2402199. 10.1002/adhm.202402199 39300854 PMC11671291

[B18] Chen Z. Zhang H. Huang J. Weng W. Geng Z. Li M. (2025). DNA-encoded dynamic hydrogels for 3D bioprinted cartilage organoids. Mater Today Bio 31, 101509. 10.1016/j.mtbio.2025.101509 PMC11803226 39925718

[B19] Cho A. N. Jin Y. An Y. Kim J. Choi Y. S. Lee J. S. (2021). Microfluidic device with brain extracellular matrix promotes structural and functional maturation of human brain organoids. Nat. Commun. 12 (1), 4730. 10.1038/s41467-021-24775-5 34354063 PMC8342542

[B20] Cho Y. You J. Lee J. H. (2025). Natural polymer-based hydrogel platforms for organoid and microphysiological systems: mechanistic insights and translational perspectives. Polymers 17 (15), 2109. 10.3390/polym17152109 40808156 PMC12349552

[B21] Chrisnandy A. Lutolf M. P. (2025). An extracellular matrix niche secreted by epithelial cells drives intestinal organoid formation. Dev. Cell 60 (22), 3116–3130.e8. 10.1016/j.devcel.2025.06.026 40680738

[B22] Chrisnandy A. Blondel D. Rezakhani S. Broguiere N. Lutolf M. P. (2022). Synthetic dynamic hydrogels promote degradation-independent *in vitro* organogenesis. Nat. Mater 21 (4), 479–487. 10.1038/s41563-021-01136-7 34782747

[B23] Claxton N. L. Luse M. A. Isakson B. E. Highley C. B. (2024). Engineering granular hydrogels without interparticle cross-linking to support multicellular organization. ACS Biomater. Sci. Eng. 10 (12), 7594–7605. 10.1021/acsbiomaterials.4c01563 39585331 PMC11632665

[B24] Cruz-Acuña R. Quirós M. Farkas A. E. Dedhia P. H. Huang S. Siuda D. (2017). Synthetic hydrogels for human intestinal organoid generation and colonic wound repair. Nat. Cell Biol. 19 (11), 1326–1335. 10.1038/ncb3632 29058719 PMC5664213

[B25] De Lorenzi F. Hansen N. Theek B. Daware R. Motta A. Breuel S. (2024). Engineering mesoscopic 3D tumor models with a self-organizing vascularized matrix. Adv. Mater 36 (5), e2303196. 10.1002/adma.202303196 37865947

[B26] Douillet C. Nicodeme M. Hermant L. Bergeron V. Guillemot F. Fricain J. C. (2022). From local to global matrix organization by fibroblasts: a 4D laser-assisted bioprinting approach. Biofabrication 14 (2), 025006. 10.1088/1758-5090/ac40ed 34875632

[B27] Dupont S. Morsut L. Aragona M. Enzo E. Giulitti S. Cordenonsi M. (2011). Role of YAP/TAZ in mechanotransduction. Nature 474 (7350), 179–183. 10.1038/nature10137 21654799

[B28] Edri S. Newman Frisch A. Safina D. Machour M. Zavin J. Landsman L. (2024). 3D bioprinting of multicellular stem cell-derived constructs to model pancreatic cell differentiation. Adv. Funct. Mater. 34 (30), 2315488. 10.1002/adfm.202315488

[B29] Eiken M. K. Childs C. J. Brastrom L. K. Frum T. Plaster E. M. Ahmed D. W. (2025). Nascent matrix deposition supports alveolar organoid formation from aggregates in synthetic hydrogels. Stem Cell Rep. 20 (1), 102376. 10.1016/j.stemcr.2024.11.006 PMC11784465 39672155

[B30] Giobbe G. G. Crowley C. Luni C. Campinoti S. Khedr M. Kretzschmar K. (2019). Extracellular matrix hydrogel derived from decellularized tissues enables endodermal organoid culture. Nat. Commun. 10 (1), 5658. 10.1038/s41467-019-13605-4 31827102 PMC6906306

[B31] Gjorevski N. Sachs N. Manfrin A. Giger S. Bragina M. E. Ordóñez-Morán P. (2016). Designer matrices for intestinal stem cell and organoid culture. Nature 539 (7630), 560–564. 10.1038/nature20168 27851739

[B32] Gjorevski N. Nikolaev M. Brown T. E. Mitrofanova O. Brandenberg N. DelRio F. W. (2022). Tissue geometry drives deterministic organoid patterning. Science 375 (6576), eaaw9021. 10.1126/science.aaw9021 34990240 PMC9131435

[B33] Griffin D. R. Weaver W. M. Scumpia P. O. Di Carlo D. Segura T. (2015). Accelerated wound healing by injectable microporous gel scaffolds assembled from annealed building blocks. Nat. Mater. 14 (7), 737–744. 10.1038/nmat4294 26030305 PMC4615579

[B34] He Z. Dony L. Fleck J. S. Szałata A. Li K. X. Slišković I. (2024). An integrated transcriptomic cell atlas of human neural organoids. Nature 635 (8039), 690–698. 10.1038/s41586-024-08172-8 39567792 PMC11578878

[B35] Homan K. A. Kolesky D. B. Skylar-Scott M. A. Herrmann J. Obuobi H. Moisan A. (2016). Bioprinting of 3D convoluted renal proximal tubules on perfusable chips. Sci. Rep. 6 (1), 34845. 10.1038/srep34845 27725720 PMC5057112

[B36] Huang J. Jia S. Liang R. Li A. Li L. Wang H. (2025). Construction of organoids using bioprinting technology: a frontier exploration of cartilage repair. J. Orthop. Transl. 54, 37–50. 10.1016/j.jot.2025.06.020 PMC12283559 40703568

[B37] Huang Y. Zhang X. Zhang W. Tang J. Liu J. (2025). Rational design matrix materials for organoid development and application in biomedicine. Regen. Biomater. 12, rbaf038. 10.1093/rb/rbaf038 40556786 PMC12187070

[B38] Hughes C. S. Postovit L. M. Lajoie G. A. (2010). Matrigel: a complex protein mixture required for optimal growth of cell culture. Proteomics 10 (9), 1886–1890. 10.1002/pmic.200900758 20162561

[B39] Hunt D. R. Klett K. C. Mascharak S. Wang H. Gong D. Lou J. (2021). Engineered matrices enable the culture of human patient-derived intestinal organoids. Adv. Sci. (Weinh) 8 (10), 2004705. 10.1002/advs.202004705 34026461 PMC8132048

[B40] Jeon E. Y. Sorrells L. Abaci H. E. (2022). Biomaterials and bioengineering to guide tissue morphogenesis in epithelial organoids. Front. Bioeng. Biotechnol. 10, 1038277. 10.3389/fbioe.2022.1038277 36466337 PMC9712807

[B41] Joshi A. Agrawal A. Choudhury S. Rath S. N. Joshi A. Taori K. (2025). From microparticles to bulk hydrogels: emerging granular hydrogels in cartilage tissue engineering. Biomater. Sci. 13 (18), 4916–4951. 10.1039/d5bm00801h 40765258

[B42] Khanna A. Ayan B. Undieh A. A. Yang Y. P. Huang N. F. (2022). Advances in three-dimensional bioprinted stem cell-based tissue engineering for cardiovascular regeneration. J. Mol. Cell Cardiol. 169, 13–27. 10.1016/j.yjmcc.2022.04.017 35569213 PMC9385403

[B43] Kharkar P. M. Kiick K. L. Kloxin A. M. (2013). Designing degradable hydrogels for orthogonal control of cell microenvironments. Chem. Soc. Rev. 42 (17), 7335–7372. 10.1039/c3cs60040h 23609001 PMC3762890

[B44] Kim J. W. Nam S. A. Yi J. Kim J. Y. Lee J. Y. Park S. (2022a). Kidney decellularized extracellular matrix enhanced the vascularization and maturation of human kidney organoids. Adv. Sci. (Weinh) 9 (15), e2103526. 10.1002/advs.202103526 35322595 PMC9130892

[B45] Kim S. Min S. Choi Y. S. Jo S. H. Jung J. H. Han K. (2022b). Tissue extracellular matrix hydrogels as alternatives to matrigel for culturing gastrointestinal organoids. Nat. Commun. 13 (1), 1692. 10.1038/s41467-022-29279-4 35354790 PMC8967832

[B46] Kim M. Cho S. Hwang D. G. Shim I. K. Kim S. C. Jang J. (2025). Bioprinting of bespoke islet-specific niches to promote maturation of stem cell-derived islets. Nat. Commun. 16 (1), 1430. 10.1038/s41467-025-56665-5 39920133 PMC11805982

[B47] Klasen L. Mork M. Nasehi R. Krishnamurthy V. T. Zeevaert K. Babendreyer A. (2025). Microgels enable iPSCs to assemble, expand, and differentiate into organoids — from sizable to high throughput. bioRxiv, 2025. 10.1101/2025.10.20.683388

[B48] Kozlowski M. T. Crook C. J. Ku H. T. (2021). Towards organoid culture without matrigel. Commun. Biol. 4 (1), 1387. 10.1038/s42003-021-02910-8 34893703 PMC8664924

[B49] Kratochvil M. J. Seymour A. J. Li T. L. Paşca S. P. Kuo C. J. Heilshorn S. C. (2019). Engineered materials for organoid systems. Nat. Rev. Mater. 4 (9), 606–622. 10.1038/s41578-019-0129-9 33552558 PMC7864216

[B50] Lai J. Liu Y. Lu G. Yung P. Wang X. Tuan R. S. (2024). 4D bioprinting of programmed dynamic tissues. Bioact. Mater 37, 348–377. 10.1016/j.bioactmat.2024.03.033 38694766 PMC11061618

[B51] Lai W. Geliang H. Bin X. Wang W. (2025). Effects of hydrogel stiffness and viscoelasticity on organoid culture: a comprehensive review. Mol. Med. 31 (1), 83. 10.1186/s10020-025-01131-7 40033190 PMC11877758

[B52] Lancaster M. A. Renner M. Martin C. A. Wenzel D. Bicknell L. S. Hurles M. E. (2013). Cerebral organoids model human brain development and microcephaly. Nature 501 (7467), 373–379. 10.1038/nature12517 23995685 PMC3817409

[B53] Lavickova B. Kronabitter H. Cervera‐Negueruela M. Ceylan E. Benito‐Zarza L. López‐Sandoval R. (2026). Integrated microfluidic platform for high-throughput generation of intestinal organoids in hydrogel droplets. Adv. Sci. (Weinh) 13 (13), e16507. 10.1002/advs.202516507 41491998 PMC12955914

[B54] Lawlor K. T. Vanslambrouck J. M. Higgins J. W. Chambon A. Bishard K. Arndt D. (2021). Cellular extrusion bioprinting improves kidney organoid reproducibility and conformation. Nat. Mater 20 (2), 260–271. 10.1038/s41563-020-00853-9 33230326 PMC7855371

[B55] Layrolle P. Payoux P. Chavanas S. (2022). Message in a scaffold: natural biomaterials for three-dimensional (3D) bioprinting of human brain organoids. Biomolecules 13 (1), 25. 10.3390/biom13010025 36671410 PMC9855696

[B56] Li C. An N. Song Q. Hu Y. Yin W. Wang Q. (2024a). Enhancing organoid culture: harnessing the potential of decellularized extracellular matrix hydrogels for mimicking microenvironments. J. Biomed. Sci. 31 (1), 96. 10.1186/s12929-024-01086-7 39334251 PMC11429032

[B57] Li Z. Yu D. Zhou C. Wang F. Lu K. Liu Y. (2024b). Engineering vascularised organoid-on-a-chip: strategies, advances and future perspectives. Biomater. Transl. 5 (1), 21–32. 10.12336/biomatertransl.2024.01.003 39220668 PMC11362354

[B58] Li Z. Li K. Zhang C. Zhao Y. Guo Y. He J. (2025). Bioprinted organoids: an innovative engine in biomedicine. Adv. Sci. (Weinh) 12 (33), e07317. 10.1002/advs.202507317 40712147 PMC12412588

[B59] Limasale Y. D. P. Fusenig M. Samulowitz M. Atallah P. Sievers J. Dennison N. (2024). Glycosaminoglycan concentration and sulfation patterns of biohybrid polymer matrices direct microvascular network formation and stability. Adv. Funct. Mater. 34 (49), 2411475. 10.1002/adfm.202411475

[B60] Ma W. Yang Z. Huang J. Huang J. Lu M. Ma H. (2025). Silicate biomaterials-induced bone marrow organoids for tissue regeneration. Interdiscip. Mater. 4 (6), 881–899. 10.1002/idm2.70020

[B61] Maramraju S. Kowalczewski A. Kaza A. Liu X. Singaraju J. P. Albert M. V. (2024). AI-organoid integrated systems for biomedical studies and applications. Bioeng. Transl. Med. 9 (2), e10641. 10.1002/btm2.10641 38435826 PMC10905559

[B62] Maschmeyer I. Lorenz A. K. Schimek K. Hasenberg T. Ramme A. P. Hübner J. (2015). A four-organ-chip for interconnected long-term co-culture of human intestine, liver, skin and kidney equivalents. Lab. Chip 15 (12), 2688–2699. 10.1039/c5lc00392j 25996126

[B63] Mierke C. T. (2024). Bioprinting of cells, organoids and Organs-on-a-Chip together with hydrogels improves structural and mechanical cues. Cells 13 (19), 1638. 10.3390/cells13191638 39404401 PMC11476109

[B64] Mulero-Russe A. García A. J. (2024). Engineered synthetic matrices for human intestinal organoid culture and therapeutic delivery. Adv. Mater 36 (9), e2307678. 10.1002/adma.202307678 37987171 PMC10922691

[B65] Nakamura K. Di Caprio N. Burdick J. A. (2024). Engineered shape-morphing transitions in hydrogels through suspension Bath printing of temperature-responsive granular hydrogel inks. Adv. Mater 36 (47), 2410661. 10.1002/adma.202410661 PMC11588557 39358935

[B66] Neumann M. di Marco G. Iudin D. Viola M. van Nostrum C. F. van Ravensteijn B. G. P. (2023). Stimuli-responsive hydrogels: the dynamic smart biomaterials of tomorrow. Macromolecules 56 (21), 8377–8392. 10.1021/acs.macromol.3c00967 38024154 PMC10653276

[B67] Nikolaev M. Mitrofanova O. Broguiere N. Geraldo S. Dutta D. Tabata Y. (2020). Homeostatic mini-intestines through scaffold-guided organoid morphogenesis. Nature 585 (7826), 574–578. 10.1038/s41586-020-2724-8 32939089

[B68] Novak R. Ingram M. Marquez S. Das D. Delahanty A. Herland A. (2020). Robotic fluidic coupling and interrogation of multiple vascularized organ chips. Nat. Biomed. Eng. 4 (4), 407–420. 10.1038/s41551-019-0497-x 31988458 PMC8057865

[B69] Park T. Kim T. K. Han Y. D. Kim K. A. Kim H. Kim H. S. (2023). Development of a deep learning based image processing tool for enhanced organoid analysis. Sci. Rep. 13 (1), 19841. 10.1038/s41598-023-46485-2 37963925 PMC10646080

[B70] Peng Y.-H. Hsiao S. K. Gupta K. Ruland A. Auernhammer G. K. Maitz M. F. (2023). Dynamic matrices with DNA-encoded viscoelasticity for cell and organoid culture. Nat. Nanotechnol. 18 (12), 1463–1473. 10.1038/s41565-023-01483-3 37550574 PMC10716043

[B71] Pramanick A. Chakraborty J. Kennedy O. Wong H. W. Kianersi S. Kelly D. (2026). Developmentally inspired bioprinting of nascent multicellular human heart tissue through *in situ* differentiation and morphogenesis of iPSCs. Adv. Sci. 13 (45), e22241. 10.1002/advs.202522241 PMC13336075 42159371

[B72] Qazi T. H. Burdick J. A. (2021). Granular hydrogels for endogenous tissue repair. Biomater. Biosyst. 1, 100008. 10.1016/j.bbiosy.2021.100008 36825161 PMC9934473

[B73] Qazi T. H. Blatchley M. R. Davidson M. D. Yavitt F. M. Cooke M. E. Anseth K. S. (2022). Programming hydrogels to probe spatiotemporal cell biology. Cell Stem Cell 29 (5), 678–691. 10.1016/j.stem.2022.03.013 35413278 PMC9081204

[B74] Qin Y. Li J. Heng Y. Wang Z. Wu D. Rahman M. (2025). A knowledge-driven deep learning framework for organoid morphological segmentation and characterization. BMC Biol. 23 (1), 313. 10.1186/s12915-025-02411-8 41121276 PMC12538981

[B75] Quintard C. Tubbs E. Jonsson G. Jiao J. Wang J. Werschler N. (2024). A microfluidic platform integrating functional vascularized organoids-on-chip. Nat. Commun. 15 (1), 1452. 10.1038/s41467-024-45710-4 38365780 PMC10873332

[B76] Ramírez-González G. A. Consumi-Tubito C. Vargas-Méndez E. Centeno-Cerdas C. (2025). Advancing Organ-on-a-Chip systems: the role of scaffold materials and coatings in engineering cell microenvironment. Polym. (Basel) 17 (9), 1263. 10.3390/polym17091263 PMC12074455 40363048

[B77] Reginensi D. Ortiz D. A. Denis B. Castillo S. Burillo A. Khoury N. (2025). Region-specific brain decellularized extracellular matrix promotes cell recovery in an *in vitro* model of stroke. Sci. Rep. 15 (1), 11921. 10.1038/s41598-025-95656-w 40195414 PMC11976941

[B78] Rijns L. Wijnakker J. A. P. M. Veenbrink V. A. Bellan R. Craenmehr F. W. B. Hendrikse S. I. S. (2026). Controlling intestinal organoid polarity using synthetic dynamic hydrogels decorated with laminin-derived IKVAV peptides. Adv. Healthc. Mater. 15, e02079. 10.1002/adhm.202502079 40904064 PMC12927536

[B79] Rozenblatt-Rosen O. Stubbington M. J. T. Regev A. Teichmann S. A. (2017). The human cell atlas: from vision to reality. Nature 550 (7677), 451–453. 10.1038/550451a 29072289

[B80] Ruiter F. A. A. Morgan F. L. C. Roumans N. Schumacher A. Slaats G. G. Moroni L. (2022). Soft, dynamic hydrogel confinement improves kidney organoid lumen morphology and reduces epithelial-mesenchymal transition in culture. Adv. Sci. (Weinh) 9 (20), e2200543. 10.1002/advs.202200543 35567354 PMC9284132

[B81] Sato T. Vries R. G. Snippert H. J. van de Wetering M. Barker N. Stange D. E. (2009). Single Lgr5 stem cells build crypt-villus structures *in vitro* without a mesenchymal niche. Nature 459 (7244), 262–265. 10.1038/nature07935 19329995

[B82] Schröter J. Deininger L. Lupse B. Richter P. Syrbe S. Mikut R. (2024). A large and diverse brain organoid dataset of 1,400 cross-laboratory images of 64 trackable brain organoids. Sci. Data 11 (1), 514. 10.1038/s41597-024-03330-z 38769371 PMC11106320

[B83] Segura T. (2024). From soft microgel assemblies to advanced healthcare materials. Adv. Healthc. Mater. 13 (25), 2402905. 10.1002/adhm.202402905 39171761

[B84] Sensi F. Spagnol G. Repetto O. D'Angelo E. Biccari A. Marangio A. (2025). Patient-derived extracellular matrix from decellularized high-grade serous ovarian carcinoma tissues as a biocompatible support for organoid growth. Transl. Oncol. 61, 102523. 10.1016/j.tranon.2025.102523 40946587 PMC12496255

[B85] Shao Y. Wang J. Jin A. Jiang S. Lei L. Liu L. (2025). Biomaterial-assisted organoid technology for disease modeling and drug screening. Mater. Today Bio 30, 101438. 10.1016/j.mtbio.2024.101438 PMC11757232 39866785

[B86] Singh A. (2017). Biomaterials innovation for next generation *ex vivo* immune tissue engineering. Biomaterials 130, 104–110. 10.1016/j.biomaterials.2017.03.015 28335993

[B87] Soliman B. G. Nguyen A. K. Gooding J. J. Kilian K. A. (2024). Advancing synthetic hydrogels through nature-inspired materials chemistry. Adv. Mater 36 (42), 2404235. 10.1002/adma.202404235 PMC11486603 38896849

[B88] Upadhyay U. Kolla S. Maredupaka S. Priya S. Srinivasulu K. Chelluri L. K. (2024). Development of an alginate-chitosan biopolymer composite with dECM bioink additive for organ-on-a-chip articular cartilage. Sci. Rep. 14 (1), 11765. 10.1038/s41598-024-62656-1 38782958 PMC11116456

[B89] Vaziri A. Maia R. Zhang P. Agresti L. Sjollema J. Shahbazi M. (2026). Granular hydrogels as modular biomaterials: from structural design to biological responses. Adv. Healthc. Mater. 15, e02462. 10.1002/adhm.202502462 41013958 PMC12817117

[B90] Wang X. Bijonowski B. M. Kurniawan N. A. (2023). Vascularizing organoids to promote long-term organogenesis on a chip. Organoids 2 (4), 239–255. 10.3390/organoids2040019

[B91] Wang J. Wu Y. Li G. Zhou F. Wu X. Wang M. (2024a). Engineering large-scale self-mineralizing bone organoids with bone matrix-inspired hydroxyapatite hybrid bioinks. Adv. Mater 36 (30), 2309875. 10.1002/adma.202309875 38642033

[B92] Wang H. Li X. You X. Zhao G. (2024b). Harnessing the power of artificial intelligence for human living organoid research. Bioact. Mater 42, 140–164. 10.1016/j.bioactmat.2024.08.027 39280585 PMC11402070

[B93] Wang Q. Yuan F. Zuo X. Li M. (2025). Breakthroughs and challenges of organoid models for assessing cancer immunotherapy: a cutting-edge tool for advancing personalised treatments. Cell Death Discov. 11 (1), 222. 10.1038/s41420-025-02505-w 40335487 PMC12059183

[B94] Wijnakker J. van Son G. J. Krueger D. van de Wetering W. J. Lopez-Iglesias C. Schreurs R. (2025). Integrin-activating yersinia protein Invasin sustains long-term expansion of primary epithelial cells as 2D organoid sheets. Proc. Natl. Acad. Sci. U. S. A. 122 (1), e2420595121. 10.1073/pnas.2420595121 39793062 PMC11725944

[B95] Wu X. Hu Y. Sheng S. Yang H. Li Z. Han Q. (2025). DNA-based hydrogels for bone regeneration: a promising tool for bone organoids. Mater Today Bio 31, 101502. 10.1016/j.mtbio.2025.101502 39911372 PMC11795821

[B96] Xie X. Chen X. Zhou J. Wang T. Yang G. Han F. (2025). Dynamic hydrogels with tunable mechanics for 3D organoid derivation. Small 21 (29), 2501862. 10.1002/smll.202501862 40434214

[B97] Yi S. A. Zhang Y. Rathnam C. Pongkulapa T. Lee K. (2021). Bioengineering approaches for the advanced organoid research. Adv. Mater 33 (45), e2007949. 10.1002/adma.202007949 34561899 PMC8682947

[B98] Zhao Y. Landau S. Okhovatian S. Liu C. Lu R. X. Z. Lai B. F. L. (2024). Integrating organoids and organ-on-a-chip devices. Nat. Rev. Bioeng. 2 (7), 588–608. 10.1038/s44222-024-00207-z

[B99] Zhang S. Wan Z. Kamm R. D. (2021). Vascularized organoids on a chip: strategies for engineering organoids with functional vasculature. Lab. Chip 21 (3), 473–488. 10.1039/d0lc01186j 33480945 PMC8283929

[B100] Zhang Z. O'Laughlin R. Song H. Ming G. l. (2022). Patterning of brain organoids derived from human pluripotent stem cells. Curr. Opin. Neurobiol. 74, 102536. 10.1016/j.conb.2022.102536 35405627 PMC9167774

[B101] Zhang T. Sheng S. Cai W. Yang H. Li J. Niu L. (2024). 3-D bioprinted human-derived skin organoids accelerate full-thickness skin defects repair. Bioact. Mater 42, 257–269. 10.1016/j.bioactmat.2024.08.036 39285913 PMC11404058

[B102] Zhao Z. Chen X. Dowbaj A. M. Sljukic A. Bratlie K. Lin L. (2022). Organoids. Nat. Rev. Methods Prim. 2 (1), 94. 10.1038/s43586-022-00174-y PMC10270325 37325195

